# Increased Breeding Frequency Mitigates Inbreeding Depression in Peromyscus in Captivity

**DOI:** 10.1002/ece3.71728

**Published:** 2025-07-10

**Authors:** Kim‐Tuyen Huynh‐Dam, Celia Jaeger, Angeliki Tsomos, Debra Norman, Hippokratis Kiaris

**Affiliations:** ^1^ Department of Drug Discovery and Biomedical Sciences, College of Pharmacy University of South Carolina Columbia South Carolina USA; ^2^ Peromyscus Genetic Stock Center University of South Carolina Columbia South Carolina USA

**Keywords:** breeding frequency, breeding performance, closed colony, deer mice, inbred, inbreeding depression, Peromyscus

## Abstract

Increased parental relatedness occurs in small wild populations and in closed colonies in captivity and reduces offspring fitness. A closed colony of *Peromyscus maniculatus* is maintained as genetically diverse stock at the Peromyscus Genetic Stock Center since 1963. Breeding records are available for all the years of breeding in captivity, which allows evaluation of the breeding performance since the inception of the stock. Kinship calculations showed that increased parental relatedness results in offspring loss, which is consistent with the operation of inbreeding depression and is common in small populations, both wild and captive. Nonetheless, an adaptive response was recorded that mitigated the adverse consequences of inbreeding and contributed to the long‐term stability of the colony: When parental relatedness increased, more offspring were produced, resulting in the overall number of viable offspring being unaffected. The underlying mechanism involved adjustments in the interval for mating between related parents, causing the production of more litters. These adaptive changes indicate that the harmful consequences of inbreeding may be partially relieved by mechanisms involving changes in the animals' reproductive strategy. The availability of the breeding records of *P. maniculatus* enables the performance of additional studies asking different questions regarding the breeding dynamics of a closed colony under regulated conditions.

## Graphical abstract

Analysis of the breeding records of *Peromyscus maniculatus* in captivity indicated the operation of inbreeding depression that triggered an adaptive response according to which when parental relatedness increased, more offspring are produced. The underlying mechanism involved adjustments in the interval for mating between related parents causing the production of more litters. These adaptive changes indicate that the harmful consequences of inbreeding may be partially relieved by mechanisms involving changes in the animals' reproductive strategy.

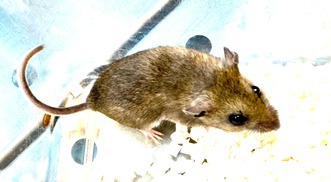

## 1 Introduction

Small populations in nature and in closed colonies in captivity are prone to inbreeding depression because deleterious alleles are reduced to homozygosity (Charlesworth and Willis 2009; Huisman et al. 2016). Yet, several small communities around the world, due to their geographical and cultural isolation, or due to assortative matings, are characterized by increased longevity and favorable health outcomes despite the fact that small population size favors inbreeding (Mitchell et al. 2001; Panagiotakos et al. 2011; Buettner and Skemp 2016; Robinson et al. 2017). In addition, closed colonies in captivity remain viable for extended periods despite the unavoidable occurrence of inbreeding depression due to the relatedness of the breeders (de Groot et al. 2025; Simpson et al. 2013; Taylor et al. 2017). Thus, compensatory mechanisms may operate that, at least in part, counterbalance the effects of inbreeding.

To explore this hypothesis, we used *Peromyscus maniculatus* (North American deer mouse) as a model that has been maintained as a closed colony since 1962 at the Peromyscus Genetic Stock Center (PGSC) (Havighorst et al. 2017). Originally, the stock (BW) originated from 40 animals caught near Ann Arbor, Michigan, in 1948. Breeding records are available for all animals born at the PGSC that, among other information, include parents' ID, date of birth, number of pups at birth, and number of male and female offspring at weaning. This allows calculation of the parental relatedness by pedigree analysis for all animals born in our facilities (Sinnwell et al. 2014). Since the inception of the stock, more than 7000 breeding records are available involving approximately 33,000 animals born in captivity. The animals maintain high genetic heterogeneity as indicated by SNP analysis (Lucius et al. 2021) and retain their wild type characteristics such as seasonality in their breeding and methylation profiles (Huynh‐Dam et al. 2025; Horvath et al. 2021).

**FIGURE 1:**
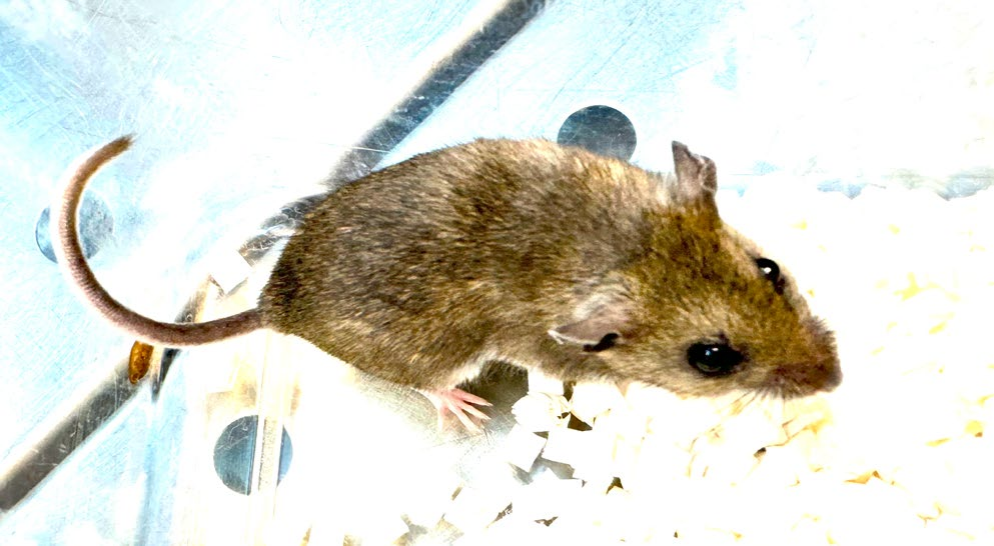
*Peromyscus maniculatus* bairdii (BW stock). BW stock is maintained at the Peromyscus Genetic Stock Center since 1960s as closed outbred colony.

By analyzing these data, we leveraged this information to unveil potential changes in the reproductive performance of *P. maniculatus* over the years in captivity (Figure 1). Our studies were prompted both by our curiosity to unveil potential changes in breeding patterns and to explore the impact of inbreeding depression due to closed breeding on the population's health. Our analyses confirmed the occurrence of inbreeding depression but also pointed to changes in breeding strategy and performance that, in part, offset the adverse consequences of inbreeding on offspring fitness.

## 2 Materials and Methods

### 2.1 Breeding

Breeding records were maintained in Microsoft Access, Microsoft Excel and hard copies. Breeding records include parental cage identities, date of birth, number of pups at birth, and number of male and female offspring at weaning. The subsequent fate of the animals, including the age and cause of death or other phenotypes of interest, was not systematically recorded because, unless the animals were used by investigators at the University of South Carolina for experimental studies, they were distributed to outside users or sacrificed if unwanted. The loss of offspring during weaning was inferred by the difference between the pups at weaning and pups at birth, and the loss of male and female offspring was calculated assuming a ratio of male: female pups at birth of 1:1. For all years of PGSC operation, breeding was performed according to institutional guidelines as required. Current IACUC is 2644‐101795‐042823. Animals are kept in a 16 h light/8 h dark cycle under standard animal facility conditions. Breeding pairs for colony maintenance were established by animals older than 2 months. Throughout the operation of the PGSC, colony managers aimed to maintain genetic diversity, and therefore closely related animals were not selected as breeders. Breeders were typically replaced after 1.5–2 years or when their productivity was reduced.

### 2.2 Data Collection and Preprocessing

The dataset for this study comprised breeding records from *Peromyscus* species, specifically the BW stock, spanning multiple years of controlled breeding. Raw data included details on litter birthdates, weaning status, sex distribution, mortality, and parental identities (Dam and Sire) for each breeding cage (referred to as *Mating Number*).

Data cleaning steps involved:

- Removing duplicate entries.
- Filtering out cases with incomplete or inconsistent records for stock, mating numbers, birth, and weaning data.
- Capping weaning counts to match birth counts to account for potential data entry errors.
- Calculating the number of dead mice (*Dead mice*) as the difference between the total number of mice born and weaned.

The initial number of breeding pairs in the dataset was 1616, identified by unique *Mating Number* entries. After quality control filtering and the exclusion of invalid or incomplete records, the final analysis included 1312 breeding pairs, with 304 pairs removed due to data quality concerns. These breeding pairs together contributed a total of 7858 individual birth records, each representing a distinct litter or reproductive event.

**FIGURE 2:**
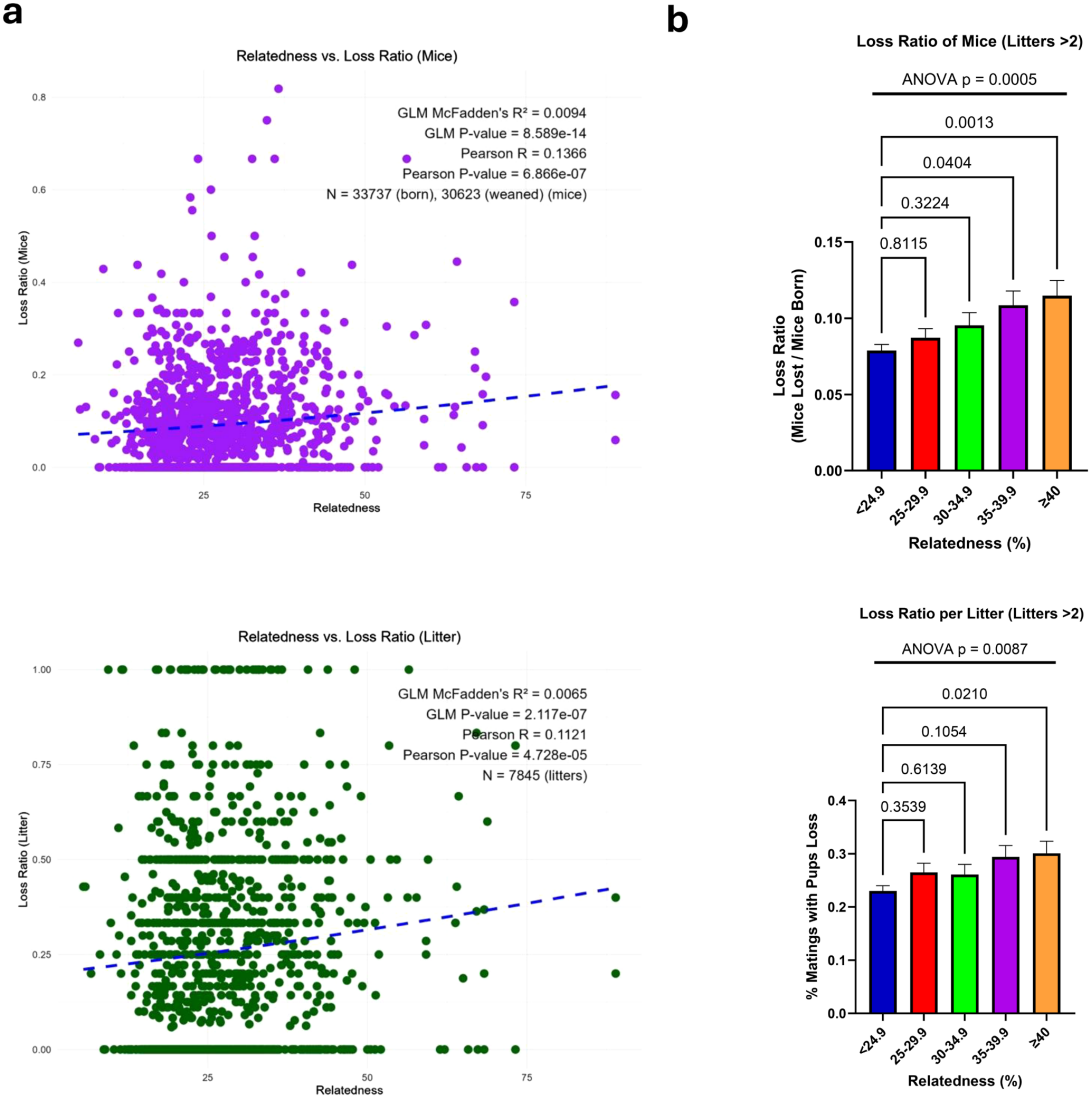
Inbreeding depression in *Peromyscus maniculatus* . (a) Parental relatedness vs. ratio of offspring lost until weaning (upper panel) or ratio of litters at which offspring loss has been recorded until weaning (lower panel). Ratio of offspring lost until weaning is the fraction of offspring lost per total mice born. Ratio of litters at which offspring loss has been recorded is the fraction of the litters at which loss of offspring had occurred until the weaning per total litters. (b) Ratio of mice lost until weaning (upper panel) or ratio of litters at which offspring loss has been recorded (lower panel) in different cohorts of relatedness. For ratio calculations breeding pairs that produced more than 2 litters were used to allow average litter calculations. Statistical analyses were performed by Pearson's *R* and McFadden's *R* ^2^ (a) or ANOVA (b). *p* values are shown.

For pedigree analysis, kinship was calculated by analyzing five preceding generations of breeding as described by Sinnwell et al. (2014). Higher numbers of generations were found to introduce errors in the calculations due to occasional ambiguous or inconsistent entries, particularly in older records. Thus, a maximum of five generations was selected to optimize data accuracy and minimize computation errors. All litters from all breeders were included in the analysis unless otherwise stated. In Figure 2, bar plots depict data filtered to include animals that produced more than two litters to enable accurate average litter loss calculations.

### 2.3 Pedigree and Relatedness Calculation

To assess genetic relatedness between Dam and Sire in each breeding pair, pedigree information was extracted from the dataset, including the parental lineage across multiple generations. The pedigree construction utilized the *kinship2* package in R, which allows for calculating the kinship coefficient (Sinnwell et al. 2014). The relatedness score was computed as twice the kinship coefficient, representing the proportion of alleles expected to be shared between the Dam and Sire. Relatedness values are expressed as percentages ranging from 0 (no shared ancestry) to 100 (genetic identity).

Each breeding cage was analyzed to extract the parental identities, and recursive ancestor tracing was performed up to five generations. The kinship matrix was then computed, and the relatedness score for each mating pair was extracted. This information was later merged with reproductive outcome data for subsequent analyses.

### 2.4 Reproductive Analysis and Statistical Modeling

Reproductive success was quantified in several ways:

1. Loss ratio per litter: Calculated as the proportion of litters where at least one pup was lost.
2. Loss ratio by total mice: Calculated as the number of dead mice divided by the total number of mice born.
3. Interval between mating and first litter: Computed as the number of days between the recorded mating date and the birth of the first litter.
4. Delivery interval: The time in days between consecutive litters within the same mating cage.

Data were analyzed using Generalized Linear Models (GLMs) with a binomial distribution and logit link function, as the outcome variables (loss ratios) are proportions. GLMs were weighted by the total number of events (litters or mice born) to adjust for varying litter sizes across breeding cages. For model evaluation, both McFadden's *R* ^2^ and Pearson's *R* correlation coefficients were computed. McFadden's *R* ^2^, a metric specific to logistic regression, evaluates the model's fit against a null model with no predictors, whereas Pearson's *R* assesses linear correlation strength.

Significance testing for GLM coefficients was performed using:

- Likelihood Ratio Tests (LRTs) for McFadden's *R* ^2^.
- *p* values for Pearson's *R* to evaluate the significance of correlations.

Longitudinal analyses were conducted to examine the relationship between parental relatedness and reproductive outcomes over time. These analyses spanned from 1963 to 2024 and were visualized as continuous trends in the figures presented. To account for varying breeding experiences, the analysis was performed across different thresholds of litter production, including breeders with more than 1, 2, 3, or 4 litters, with the results for these threshold analyses available in the linked GitHub repository.

### 2.5 Sex‐Based Analysis

To understand sex‐specific patterns in loss ratios, sex‐specific survival rates (male vs. female) were analyzed separately. The total number of males and females weaned was compared to the expected number based on birth data. Loss ratios were calculated independently for males and females and assessed over time using GLMs to capture trends (Nelder and Wedderburn 1972).

The temporal dynamics of these sex‐based differences were visualized with separate correlation plots for:

1. Pearson's *R* and its *p* value.
2. McFadden's *R* ^2^ and its *p* value (McFadden 1972).

These metrics were plotted over time to observe changes in correlation strength and model fit with increasing years.

### 2.6 Time‐Based Trend Analysis

Longitudinal analyses were conducted to examine the relationship between relatedness and reproductive outcomes over time. The analysis was performed for both:

- Loss ratio per litter
- Loss ratio by total mice

We calculated GLM correlations and Pearson's *R* for cumulative intervals from 1963 up to each year through 2024. To visualize temporal trends, correlation coefficients (Pearson's *R* and McFadden's *R* ^2^) and their corresponding *p* values were plotted for each cumulative interval, allowing for the identification of trends over time.

### 2.7 Grouping and Comparative Analysis

To further understand how relatedness influences reproductive success, relatedness scores were grouped into five categories:

- < 24.9%
- 25%–29.9%
- 30%–34.9%
- 35%–39.9%
- ≥ 40%

ANOVA tests were performed to identify significant differences in loss ratios across these relatedness groups. Results were visualized using bar plots, with error bars representing standard error, and ANOVA *p* values annotated directly on the plots.

### 2.8 Data Export and Visualization

All summary statistics, relatedness calculations, and GLM analysis results were exported to Excel for traceability and further inspection. High‐resolution plots were generated and saved in the ./database directory with filenames that clearly indicate the type of analysis and species involved.

The analysis was conducted using R (R Core Team 2024) and RStudio (RStudio Team 2024). The complete R scripts used for data processing, statistical analysis, and visualization are available for review at the GitHub repository: https://github.com/KimTuyenHuynhDam/Relatedness_database_pero.

## 3 Results

### 3.1 Inbreeding Depression in Closed Bred *P. maniculatus*

Initially we explored the presence of inbreeding depression by testing if the loss of offspring correlates with parental relatedness. To that end, we calculated the difference between offspring produced and weaned to estimate the number of lost offspring and expressed it in correlation with the relatedness of the parents. As shown in Figure 2, increased parental relatedness was significantly associated with loss of offspring, which confirms the operation of inbreeding depression in the colony. Furthermore, losses due to increasing parental relatedness were reflected in both the number of animals lost (Figure 2a,b, upper panels) and the fraction of the litters at which loss of offspring had occurred until the weaning of the pups (Figure 2a,b, lower panels). These associations were statistically significant, with Pearson correlations observed for both individual mice (*R* = 0.1366, *p* = 6.87e‐07) and litters (*R* = 0.1121, *p* = 4.73e‐05). Additionally, McFadden's *R* ^2^ values from logistic regression models (0.0094 for individual mice and 0.0065 for litters) further confirmed the significance of this relationship, despite explaining a relatively small portion of the variance (indicating other environmental or biological factors also contribute substantially to loss rates). Thus, not only within the same litter are more pups lost when parental relatedness increases, but also between different matings that differ in the relatedness of the mating partners, offspring loss is recorded more frequently when the relatedness of the parents is higher. These findings align with the principles of inbreeding depression, where increased fatal homozygosity associated with higher parental relatedness may contribute to reduced offspring viability.

### 3.2 Relatedness and Litter Size at Weaning Over the Years

**FIGURE 3:**
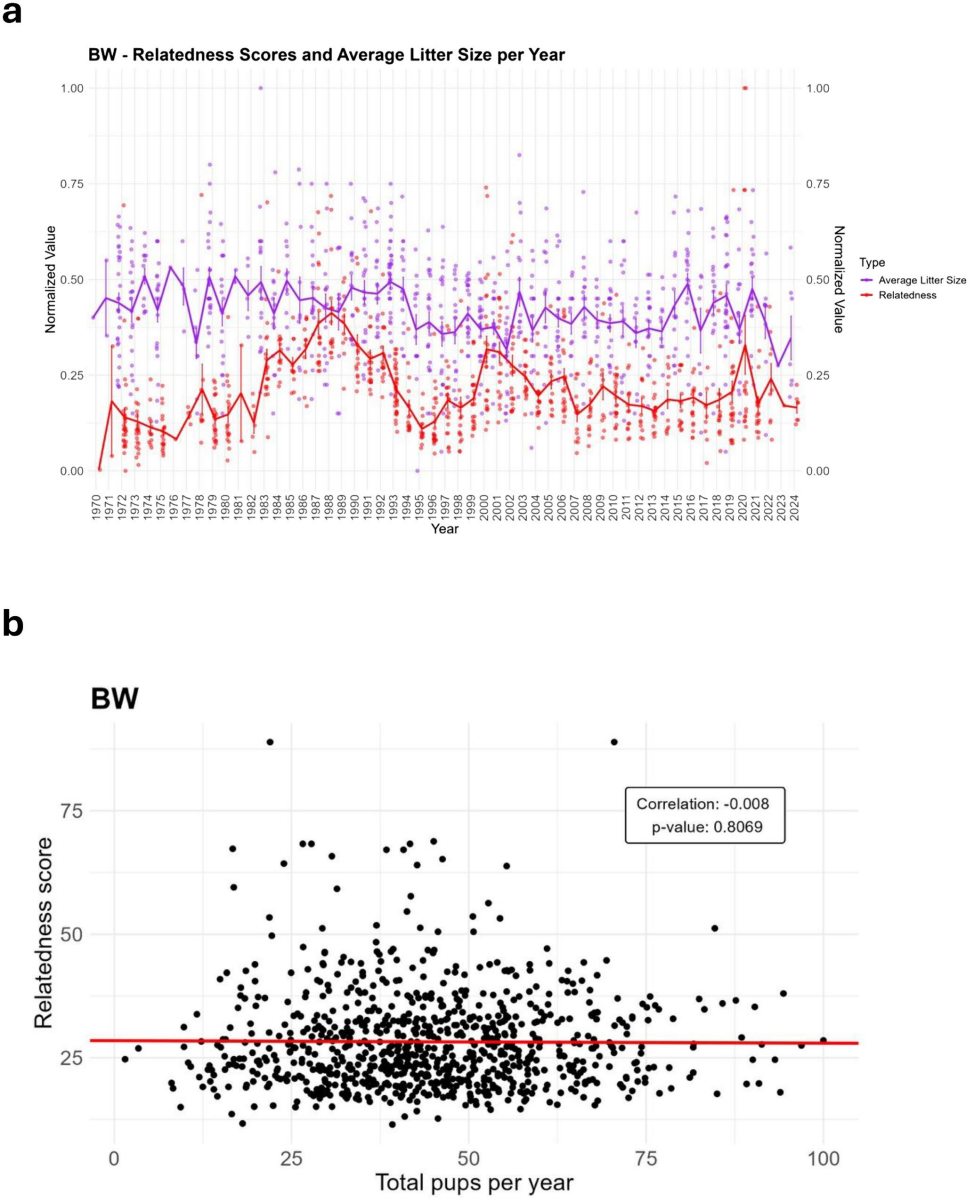
Offspring at weaning and relatedness over the years. (a) Normalized average litter sizes at weaning (purple) and relatedness (red) over the years. (b) Relatedness of parents and total pups per year at weaning. *R* (Pearson's correlation), and *p* values are shown.

Next, we asked if, since 1962, when the detailed records became available, parental relatedness and litter sizes at weaning changed, and whether they were correlated with each other. As shown in Figure 3a, the average litter size at weaning did not change considerably over the years despite the fluctuations in relatedness recorded during this period. Both normalized average litter size (purple) and relatedness scores (red) were plotted over time, showing that while relatedness scores experienced fluctuations, average litter size remained relatively stable. This stability is reflected in the absence of a strong trend across the years, highlighting the resilience of litter sizes despite the changing genetic landscape of the colony.

Furthermore, Figure 3b presents the relationship between total pups weaned per year and parental relatedness. Consistent with the observations of litter size stability, there was no significant correlation detected (*r* = −0.008, *p* = 0.8069). The near‐zero correlation coefficient and the nonsignificant *p* value indicate that parental relatedness had no detectable impact on the number of pups weaned per year throughout the colony's maintenance.

Thus, despite the occurrence of inbreeding depression, as reflected in the loss of offspring by the time of weaning (Figure 2), average litter size at weaning remained unaffected over more than 60 years of closed breeding. This suggests the operation of mechanisms that may mitigate the harmful consequences of inbreeding in offspring fitness, maintaining litter size even as relatedness fluctuated. The robustness of litter size, in spite of varying relatedness, could be attributed to selective pressures or compensatory biological mechanisms within the colony.

### 3.3 Loss of Pups and Relatedness Over the Years

To delve deeper into the assessment of reproductive performance of *P. maniculatus* in captivity, we explored whether shifts in the animals' breeding profile had occurred over the years of closed breeding or if the same trends remained consistent since 1963. Initially, we asked if the number of mice lost until weaning changed throughout the years by calculating the progressive, time‐dependent change of the correlation coefficient between parental relatedness and various breeding parameters. This approach was deemed more informative than the cumulative estimation of breeding performance (relatedness vs. total offspring, Figure 3) because it eliminates potential bias due to the unintentional selection of the mating pairs at different times. Furthermore, it enables assessment of changes in the breeding dynamics throughout the years, instead of their cumulative depiction at the end of the recording period. Finally, the consideration of the correlation coefficient between relatedness and litter loss, as opposed to cumulative numbers of offspring, factors in potential fluctuations in colony size due to uneven external demand throughout the 60‐year period of operation.

**FIGURE 4:**
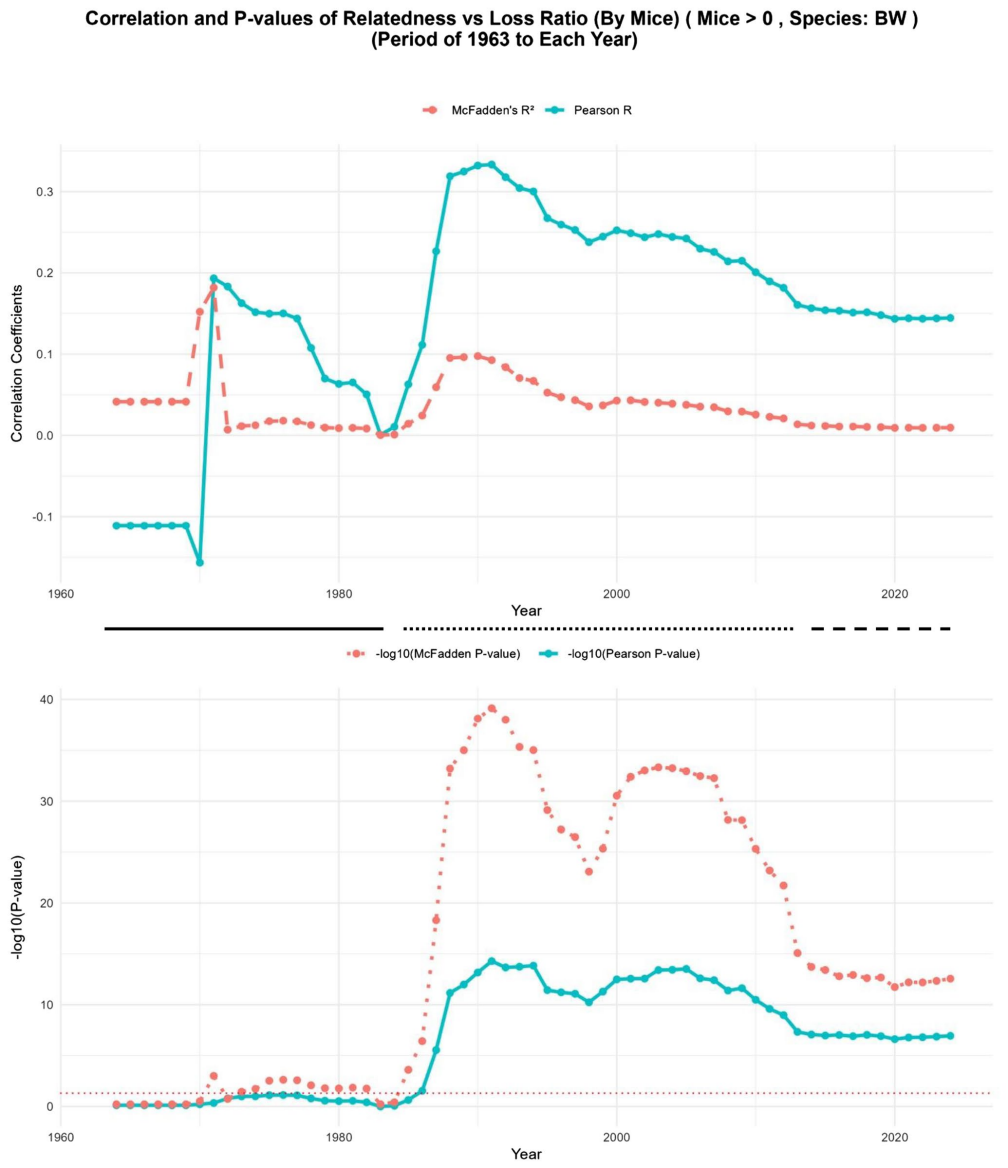
Relatedness‐dependent transition in *Peromyscus maniculatus* breeding. The upper panel shows the coefficient of correlation (McFadden's *R* ^2^, red and Pearson's *R*, green) between parental relatedness and ratio of total offspring lost. Calculations of correlation were performed at each indicated time from the inception of the stocks until the time point indicated in the *X* axis. *p* values (−log_10_) are shown in the lower panel. Dashed line shows the cutoff for significance (*p* = 0.05). The three different phases of breeding pattern (see text for details) are indicated by solid (Phase I), dotted (Phase III) and dashed (Phase III) lines.

As depicted in Figure 4 and Figure S1, the analysis revealed three distinct breeding phases in the colony's history, which were consistently observed for both loss ratio by mice and loss ratio by litters. During the initial phase (Phase I), spanning from the onset of records until approximately 1985, there was minimal association between parental relatedness and offspring loss, as indicated by both Pearson's *R* and McFadden's *R* ^2^ (Figure 4, upper panels). This lack of correlation suggests that during the early years of breeding, relatedness did not significantly impact the loss of pups.

A marked shift was observed around Phase II, starting approximately from 1985 and extending to about 2015. During this period, Pearson's *R* demonstrated a pronounced positive correlation between parental relatedness and offspring loss, with correlation coefficients peaking around 0.3. This trend was paralleled by McFadden's *R* ^2^, which, although lower in absolute terms compared to Pearson's *R*, showed a significant and stable association over this time span. The −log_10_ (*p*‐value) plots illustrate the significance of these associations, with both Pearson's *R* and McFadden's *R* ^2^ showing statistical significance (*p* < 0.05) consistently throughout Phase II (Figure 4, lower panels). These findings underline a period of strong inbreeding depression effects, where elevated relatedness between mating pairs resulted in substantially higher loss of offspring ratios.

The final Phase III, commencing around 2015 and extending to the present, is characterized by a noticeable decline in both correlation coefficients. Although Pearson's *R* remains slightly positive, it diminishes considerably, and McFadden's *R* ^2^ stabilizes near zero, indicating a potential decoupling of relatedness and offspring loss. This suggests either an adaptation mechanism within the colony or alterations in breeding management that mitigated the previous effects of inbreeding depression.

For these analyses, all available data were considered (all litters produced were used in the calculations). Similar results were obtained when data involving > 1,2, 3, or 4 litters were used (see information described in Section 2).

### 3.4 Offspring Lost at Weaning by Sex

To investigate whether there is a disparity in the loss of male versus female offspring from birth to weaning, we estimated sex‐specific loss by assuming an equal 1:1 male‐to‐female ratio at birth. The analysis was performed by comparing the number of male and female pups weaned against the assumed equal birth distribution. Although differences were recorded, they remained insignificant or occasionally reached only moderate significance.

**FIGURE 5:**
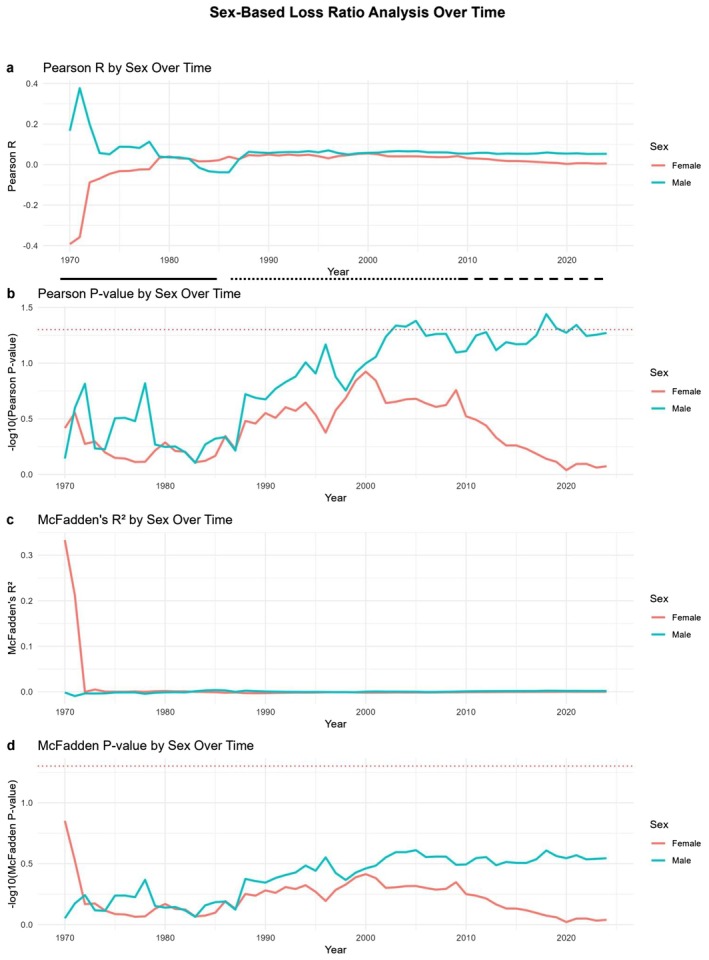
Loss of male and female offspring over the years due to parental relatedness. Coefficient of correlation (*R*, Pearson's) (a) or McFadden's *R* ^2^ (c) between relatedness and male or female offspring lost. For the calculation of male and female offspring 1:1 ratio of male:female offspring produced at birth was assumed. Calculations of correlation were performed at each indicated time from the inception of the stocks until the time point indicated in the *X* axis. *p* values (−log_10_) are shown in (b, d) as indicated. Dashed line shows the cutoff for significance (*p* = 0.05). The three different phases of breeding pattern (see text for details) are indicated by solid (Phase I), dotted (Phase III) and dashed (Phase III) lines.

As shown in Figure 5, the sex‐based analysis of loss ratios over time reveals distinct differences during the three identified breeding phases. During Phase I, the data indicate a clear, albeit insignificant, trend towards higher male offspring loss as evidenced by Pearson's *R* values (Figure 5a,b) that show a positive correlation for males and a negative correlation for females. This suggests that, relative to their assumed birth numbers, more male pups were lost during weaning compared to females.

Moving into Phase II, the sex‐specific trends begin to converge, reflecting a diminishing disparity between male and female loss ratios. Pearson's *R* values stabilize around zero for both sexes, suggesting the initial male‐biased loss rate was mitigated over time. This shift corresponds to the colony's broader transition in breeding dynamics observed in earlier analyses.

Finally, in Phase III, Pearson's *R* (Figure 5a) converges to near‐zero values for both sexes, indicating minimal correlation between parental relatedness and offspring loss for either sex. It is noted, though, that despite its low values, Pearson's *R* attained significance for the males at the late stages of Phase II that persisted during Phase III.

Notably, McFadden's *R* ^2^, which measures the model's goodness of fit for predicting loss ratios based on relatedness, remained consistently low across all periods. This further confirms that while there were historical differences in sex‐based losses, these disparities were effectively neutralized in the later years of breeding.

The observed changes may reflect adjustments in breeding management, natural selection pressures that favored more robust male offspring, or stochastic variations within the colony. The McFadden *p* values (Figure 5d) also support the lack of significant sex‐based relatedness effects in recent years, further suggesting a stabilization of sex‐specific loss ratios.

Overall, whereas initial periods of breeding showed a male‐biased loss rate, this disparity was not sustained and appears to have been corrected over time, coinciding with broader changes in breeding strategy and colony adaptation.

### 3.5 Pups Born and Pups Weaned vs Relatedness Over the Years

**FIGURE 6:**
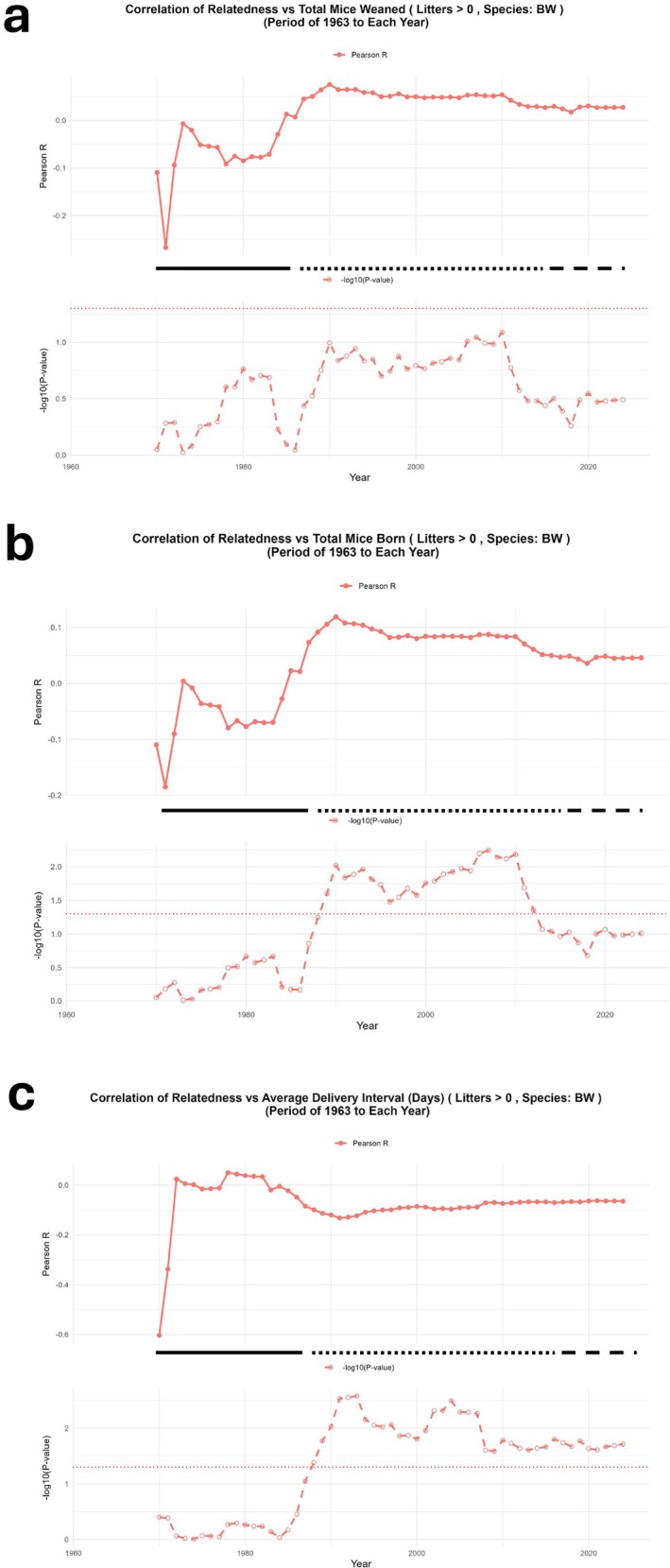
Pups born and pups weaned in association with parental relatedness over the year. The upper panels show the coefficient of correlation (*R*, Pearson's) between relatedness and mice weaned (a), mice born (b), and average delivery intervals over the years of closed breeding (c). Calculations of correlation were performed at each indicated time from the inception of the stocks until the time point indicated in the *X* axis. *p* values (−log_10_) are shown in the lower panels. Dashed line shows the cutoff for significance (*p* = 0.05). The three different phases of breeding pattern (see text for details) are indicated by solid (Phase I), dotted (Phase III) and dashed (Phase III) lines.

Subsequently, we investigated whether differences occurred over time between the number of pups born and the number of pups weaned in association with parental relatedness. As illustrated in Figure 6a,b, a clear distinction emerged in the correlation between relatedness and reproductive output over the studied periods.

In Figure 6a, the correlation between relatedness and the total number of mice weaned fluctuated considerably over time and did not reach consistent statistical significance throughout the three recorded phases. The Pearson correlation values oscillated, and the corresponding *p* values never crossed the significance threshold (−log_10_(*p*) > 1.3, equivalent to *p* < 0.05) for extended periods, indicating that the relatedness of parents did not reliably affect the number of pups weaned. These findings suggest that while offspring were produced in various numbers, their number at weaning was not consistently influenced by parental relatedness.

Conversely, Figure 6b demonstrated a consistently significant positive correlation between relatedness and the total number of pups born throughout Phase II (1988–2018). During this period, the Pearson correlation coefficient remained stably positive, and the corresponding *p* values were persistently below the significance threshold, highlighting a robust association. This consistent statistical significance (*p* < 0.05) indicates that more related parents tended to produce a higher number of pups during this adaptive phase. This phase likely reflects adjustments in breeding patterns that favored higher reproductive output in closely related pairs, potentially as a compensatory mechanism to counteract inbreeding depression effects.

The divergence in correlation patterns between pups born and pups weaned suggests that although parental relatedness contributed to higher birth rates, it did not guarantee increased survival to weaning age. The statistical evidence here clearly supports these observations, aligning with the notion that relatedness drives reproductive output but not survival to weaning. This is consistent with the results of Figure 2 indicating significant loss of offspring when parental relatedness increased, which is, however, compensated by the increased number of pups produced by more related parents (Figure 6b).

### 3.6 Changes of Breeding Frequency With Relatedness

In view of the lack of correlation between litter size at weaning and relatedness (Figure 3), we asked if the animals' breeding frequency was influenced by parental relatedness, since this could provide a mechanism that, at least in part, could explain the higher number of pups produced at Phase II. Indeed, further analysis of the breeding records showed that the higher breeding rate between related parents at Phase II was due to an adjustment in the interval between different matings, was inversely correlated with relatedness, and coincided with the period at which the association between relatedness and pups born was recorded (Figure 6b,c). This shorter interval between productive matings predicts a higher number of offspring produced by the parents that are related more. After this approximately 30‐year period, the association between relatedness and breeding was lost, indicating the acquisition of an equilibrium in the colony at which breeding became independent of relatedness (Phase III). When, instead of the interval between subsequent matings, the time for the production of the first litter was calculated, no significant association was detected during the three different periods analyzed (Figure S2a). When, however, all the breeding records were analyzed cumulatively together, a weak, albeit significant inverse correlation was found between parental relatedness and both the interval between matings and the first litter (*R* = −0.077, *p* = 0.00629) and the interval between different litters (*R* = −0.065, *p* = 0.0194) (Figure S2b).

## 4 Discussion

To explore the mechanisms that govern inbreeding depression in *P. maniculatus* in captivity, we have analyzed the breeding profile of a closed colony that has been maintained since 1962. For our analyses, instead of calculating the cumulative reproductive efficiency in association with the relatedness of the parents, we estimated the correlation coefficient between parental relatedness and pups lost, pups born, pups weaned, and time interval between matings throughout the years of closed breeding. We hypothesized that with this approach, we may be able to unveil different reproductive strategies implemented at different periods that otherwise would have been masked by the cumulative analysis. It is noted that an inherent limitation of this approach is that due to the lack of specific information throughout the PGSC's operation, only the loss of pups during weaning was considered (pups weaned minus pups born). This approach may include cannibalization of otherwise healthy pups by their parents and does not account for the causes of death or death following weaning. In addition, the unavoidable selection for the best breeders must have occurred since the offspring of these animals would be more likely to be represented in the next generation of breeders.

Our observations suggest an adaptation in the breeding of *P. maniculatus* according to which a change in breeding strategy is adopted to assist the population in coping with the harmful consequences of inbreeding. Following an initial period during which the population is not yet impacted considerably by inbreeding (Phase I), the impact of inbreeding depression becomes prominent, and loss of pups significantly correlates with parental relatedness (Phase II). At this period, an adaptive response is initiated, and more pups are produced by the animals that are more related. This is due to a reduction in the interval between the matings that offsets the effects of inbreeding. It is plausible that during this period, deleterious alleles that are responsible for the loss of pups until weaning and causing inbreeding depression are purged out of the population. Following this period (Phase II) at which a reduction of the mating intervals between more related parents occurs, this adaptive response ceases, and parental relatedness does not impact breeding performance anymore (Phase III). Consistent with this notion, inbreeding depression may be mitigated by adaptations involving changes in the animals' breeding strategy guided by parental relatedness. Whether this adaptation that occurred in Phase II reflected group selection, an epigenetic adaptation, or even unintentional selection of more productive breeders by the colony managers remains unclear; it appears, though, that its manifestations were reversible because they were not recorded during the most recent phase of breeding (Phase III).

Pending confirmation in other species and taxa, these mechanisms are expected to have decreased impact in experimental inbred populations or in diverse wild populations with low parental relatedness. However, in smaller outbred populations and in human populations that are geographically or culturally isolated, their consequences may be more pronounced (Charlesworth and Willis 2009; Robinson et al. 2017; Hoffmann and Willi 2008). Furthermore, they provide insights regarding the adaptation of wild genetically diverse populations in captivity, and may have implications on the sustainability of captive populations in closed colonies. Studies in the amphibian yellow‐bellied toad, *Bombina variegata* , showed that inbreeding is favored and that males more closely related to the female had higher mating success (Cayuela et al. 2017). The current analysis provides a paradigm of the informative value that the analyses of the breeding records of closed outbred colonies may provide. It also paves the way for similar analyses in other stocks. Furthermore, it enables additional studies asking different questions that can be answered by reanalysis of the breeding records of the BW stock of *P. maniculatus* of the PGSC.

## Author Contributions

**Kim‐Tuyen Huynh‐Dam:** data curation (equal), formal analysis (lead), investigation (equal), methodology (equal), validation (supporting), visualization (lead), writing – review and editing (equal). **Celia Jaeger:** data curation (lead), resources (lead), writing – review and editing (equal). **Angeliki Tsomos:** data curation (equal), writing – review and editing (equal). **Debra Norman:** data curation (equal), writing – review and editing (equal). **Hippokratis Kiaris:** conceptualization (equal), data curation (supporting), formal analysis (supporting), funding acquisition (lead), investigation (equal), project administration (lead), resources (lead), supervision (lead), writing – original draft (lead), writing – review and editing (equal).

## Conflicts of Interest

The authors declare no conflicts of interest.

## Supporting information


**Figure S1.** Relatedness‐dependent transition in *Peromyscus maniculatus* breeding.

**Figure S2.** Relatedness and mating time intervals.

## Data Availability

Animals and detailed breeding records are available from the PGSC. Breeding records and associated code applied in the present analysis can be found here: https://github.com/KimTuyenHuynhDam/Relatedness_database_pero.
