# Supplementary figures and images for: Increased Breeding Frequency Mitigates Inbreeding Depression in Peromyscus in Captivity
Source: Ecol Evol. 2025 Jul 10;15(7):e71728. doi: 10.1002/ece3.71728 (PMC12247253; doi:10.1002/ece3.71728)

## Slide 1
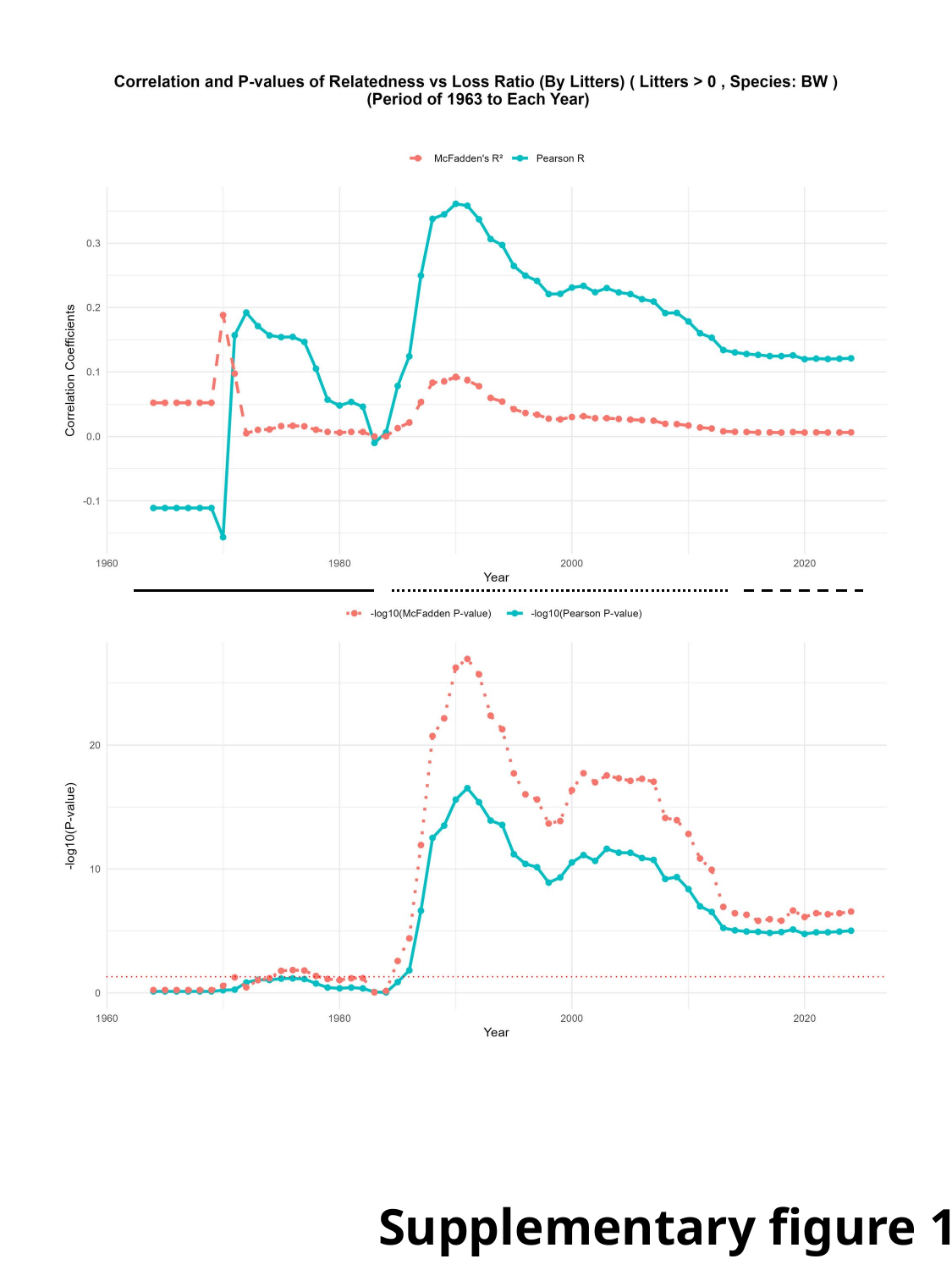

Supplementary figure 1

Supplement: Supplementary file 1 — Figure S1. Relatedness‐dependent transition in Peromyscus maniculatus breeding. [file ECE3-15-e71728-s002.pptx]

## Slide 1
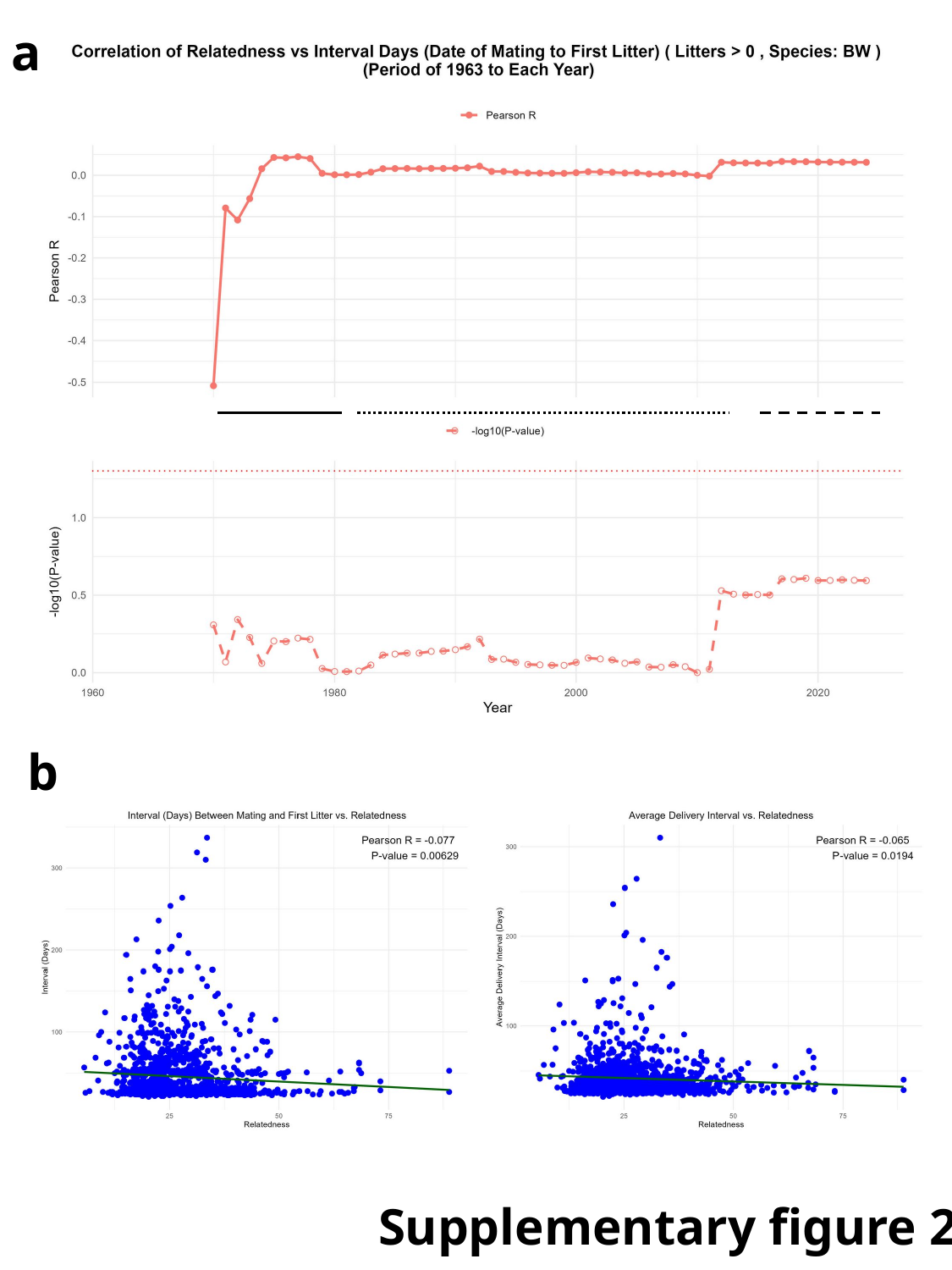

a
b
Supplementary figure 2

Supplement: Supplementary file 2 — Figure S2. Relatedness and mating time intervals. [file ECE3-15-e71728-s001.pptx]
